# Advantages of a Photodiode Detector Endoscopy System in Fluorescence-Guided Percutaneous Liver Biopsies

**DOI:** 10.3390/opt4020025

**Published:** 2023-05-15

**Authors:** Asier Marcos-Vidal, Pedram Heidari, Sheng Xu, Bradford J. Wood, Umar Mahmood

**Affiliations:** 1 Department of Radiology, Harvard Medical School, Massachusetts General Hospital, Charlestown, MA 02129, USA; 2 Center for Interventional Oncology, Radiology and Imaging Sciences, Clinical Center, National Institutes of Health, Bethesda, MD 20892, USA

**Keywords:** biopsy, ICG, fluorescence, photodetector, endoscopy

## Abstract

Image-guided liver biopsies can improve their success rate when combined with the optical detection of Indocyanine Green (ICG) fluorescence accumulated in tumors. Previous works used a camera coupled to a thin borescope to capture and quantify images from fluorescence emission during procedures; however, light-scattering prevented the formation of sharp images, and the time response for weakly fluorescent tumors was very low. Instead, replacing the camera with a photodiode detector shows an improved temporal resolution in a more compact and lighter device. This work presents the new design in a comparative study between both detection technologies, including an assessment of the temporal response and sensitivity to the presence of background fluorescence.

## 1. Introduction

Advances in radiology in recent decades have made the non-invasive diagnosis of hepatic malignancies the preferred method for the screening, assessment, and monitoring of liver disorders. Ultrasound (US), contrast-enhanced Computed Tomography (CT) and Magnetic Resonance (MR) are routinely used to detect and characterize lesions growing in the liver parenchyma; nevertheless, the specificity and sensitivity of these techniques vary dramatically with the size of the lesion, tumor differentiation grade and presence of a cirrhotic liver condition [1,2]. These factors often pose challenges for the image-based early diagnosis of liver disorders, as lesions may not have a definitive characterization under radiological exams, and, consequently, patients are often referred to a biopsy [3].

Liver biopsies allow for the histopathological evaluation of hepatic tissue samples through the percutaneous sampling of the tumor. This procedure remains the gold standard method to characterize the nature of a lesion, helping to determine the stage of disease, identify the primary location of metastatic tumors, or perform genotypic analysis. However, uncertainty in needle positioning while conducting a biopsy can lead to a false-negative result, with devastating consequences for the patient. To minimize complications and improve accuracy, biopsy procedures are often guided by imaging techniques to ensure proper needle positioning and increase confidence in the sampling location [4,5].

The use of CT and US imaging to assist the interventionist in advancing and confirming the position of the needle in percutaneous image-guided biopsies has shown limited performance when targeting small lesions (<20 mm) [5]. The lack of image contrast, heterogeneity in the parenchyma, nodular morphology, or image artifacts may hinder the identification of the tumor, increasing the uncertainty of the procedure. In addition, realtime guidance is typically achieved with US, which is a technique with a lower resolution and contrast, especially in patients with obesity.

The FDA-approved near-infrared (NIR) fluorescent agent Indocyanine Green (ICG) has traditionally been used to assess liver function, tumor labeling during resection surgeries [6,7], and, recently by this group, in liver biopsies to confirm the needle’s position [8,9]. Differences in biliary excretion of ICG between healthy and malignant liver tissue or retention in neoplastic cells lead to a higher accumulation in the latter. The resulting fluorescence emission of the tumor can be detected with a thin optical endoscope introduced through a coaxial biopsy needle cannula.

The optical confirmation of needle positioning through the detection of fluorescence from ICG in liver biopsies was achieved using a clinical cystoscope coupled to a camera [8,10]. The acquired images were weighted for the exposure time to obtain absolute-intensity pixel values at the tumor site. Although the study found significant tumor-to-healthy-liver-tissue intensity ratios at the biopsy site, diffuse and out-of-focus light prevented the formation of sharp images, precluding an assessment of structural features of the tumor [8,11,12]. In addition, fluctuations in the tumor-to-healthy-liver intensity ratio can be observed when sampling multiple locations of the lesion cross-section [8,11,12]. In hepatocellular carcinomas (HCC), only well-differentiated lesions present solid homogeneous fluorescence emission, whereas in poorly differentiated HCC and metastases, the fluorescence intensity is highly heterogeneous, often presenting rim-like emission distributions.

For these reasons, instead of relying on images, the temporal fluctuations of the detected intensity as the endoscope is advanced towards the lesion can reveal the presence of a lesion in the vicinity of the tip. However, the use of scientific cameras to perform the temporal sampling of light intensity in high-dynamic-range scenarios is unreliable, since these devices usually lack a metering system to estimate the brightness of the scene prior to the exposure. Instead, automatic exposure algorithms estimate the integration time for each image based on the intensity from previous frames, which introduces a lag until the correct value for the exposure is found. This lag causes an under-sampling of rapid intensity fluctuations, limiting the temporal resolution of the readings and introducing inaccuracies to the measured data.

In contrast, single-pixel photodetectors produce an instantaneous voltage output that is linearly correlated to the incident intensity. Furthermore, these devices can be extremely light and compact, improving the handling experience of the operator in the surgery room.

In this paper, we report a modified version of our biopsy-guidance endoscope that uses a photodiode to improve the ability to register fluorescence fluctuations in real time. The performance of the new-generation scope is compared against our previous camera-based device. We investigate the temporal response and accuracy in registering rapid light variations and assess the sensitivity to low concentrations of ICG, performance in detecting deep lesions, and robustness against the presence of background fluorescence. Additionally, we explore whether the multispectral detection of NIR I and NIR II light can retrieve distance information from lesions due to spectral changes in the optical properties of the liver.

## 2. Materials and Methods

### 2.1. Endoscope Design

**Figure 1.**
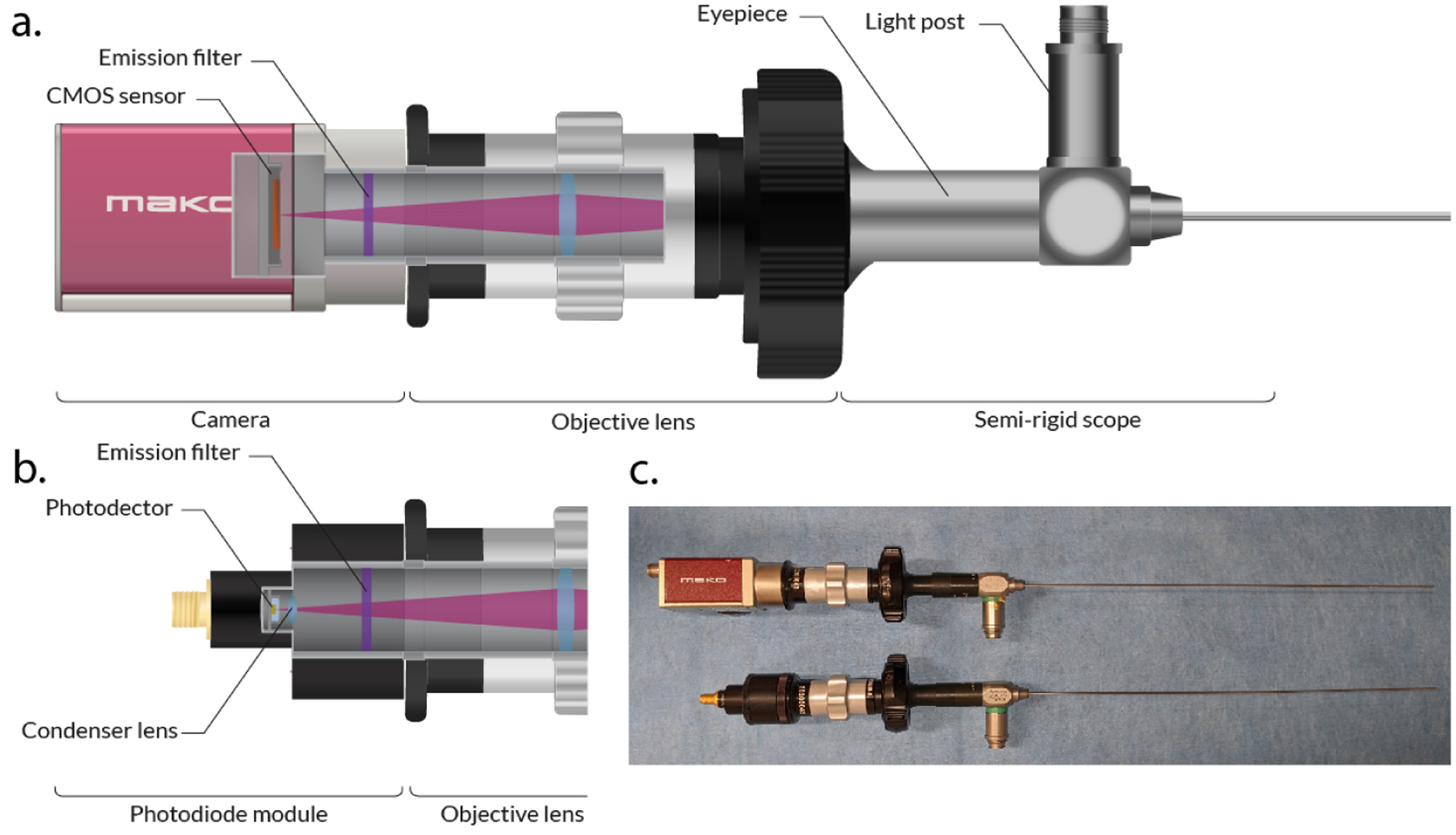
(**a**) Schematic of the camera-based endoscope. (**b**) Schematic of the photodiode-based endoscope. (**c**) Photo of both devices.

The camera-based scope hardware remains as described in our previous works [8]. Shown in Figure 1a, the system employs a clinical-grade cystoscope (27033 AA Karl Storz, Tuttlingen, Germany) to deliver the excitation light and collect the fluorescence intensity. The scope shaft has a diameter of 1.2 mm to fit through a standard 16 Gauge coaxial biopsy needle cannula. The excitation of ICG was performed using an 808 nm Coherent (Santa Clara, CA, USA) OBIS LX 150 mW laser coupled to the light post of the cystoscope.

The endoscope’s eyepiece is coupled to a Karl Storz (Tuttlingen, Germany) 38 mm c-mount objective lens to transmit the collected light to a near-infrared camera (Allied Vision Mako 223B, Exton, PA, USA) equipped with a Semrock (Lake Forest, IL, USA) 830 nm high-pass emission filter to eliminate the excitation light.

### 2.2. Photodiode-Based Endoscope Design

The new detection module, replacing the camera by a photodetector to sense ICG fluorescence light, is shown in Figure 1b. NIR I light is detected with a Si photodiode (SM05PD1A, Thorlabs Inc., Newton, NJ, USA) equipped with a Semrock 830 nm high-pass emission filter. Alternatively, fluorescence emission in the NIR II can be detected by mounting an InGaAs photodiode (SM05PD5A, Thorlabs Inc., Newton, NJ, USA) with a 1000 nm high-pass emission filter. Both detection modules have the same design and can be coupled to the objective lens of the endoscope (see Figure 1c).

The output current from the photodiode is connected to a transimpedance amplifier, and the resulting voltage signal is sampled by a DAQ (USB-6002, National Instruments, Austin, TX, USA) acquisition board.

### 2.3. Acquisition Testbench

For the tests and experiments described below, the endoscope is mounted on a micrometric-precision motorized linear stage. This allows one to control the relative position between the tip of the scope and the target.

### 2.4. Acquisition and Display Software

A custom software was developed to acquire and display the temporal evolution of the fluorescence intensity readings. The software is compatible with the camera and the photodetector modules and used throughout the experimental tests. The images from the camera and the signal from the photodetectors are read, processed and displayed in a graphical user interface developed in Java that runs over a set of C++ libraries that control the hardware. All the intensity and positioning data are saved into a timestamped log for further postprocessing.

The signal from the photodetector is sampled at 1 kHz, and the data stream is averaged with buffers of 100 samples to result in a data stream of 10 Hz. The framerate of the camera is variable since the exposure time determines the actual sampling frequency.

### 2.5. Selection of Spectral Working Window

An advantage of single-pixel detectors is the variety of substrate choices that confer different spectral responses. ICG fluorescence peaks at around 830 nm; therefore, silicon is the best option to maximize signal detection. However, the ICG emission tail in the NIR-II window can be detected by InGaAs [13–15]. Unlike cameras, InGaAs photodetectors are very affordable and thus worth exploring for investigations if the differences in the detected intensity in the NIR I and NIR II, due to changes in the optical properties, can be used to retrieve information on the distance to the emitter. Intensity readings in both spectral windows are compared to evaluate if distance information can be retrieved from the NIR I-to-NIR II ratios.

### 2.6. Performance Assessment

The performance of the photodetector detection module is evaluated against the camera version in terms of sensitivity, temporal response, and performance in detecting fluorescent lesions at multiple depths and concentrations. The metrics used to evaluate the new device include detector linearity and dynamic range, sensitivity to low concentrations of ICG, and sensitivity to distant lesions.

#### 2.6.1. Detector Linearity and Dynamic Range

The linearity and dynamic range of the sensors are evaluated by measuring the response to illumination at multiple known incident power values. The scope is illuminated with laser light at 808 nm, which is passed through a diffuser for homogenization. The incident power at the back focal plane of the objective lens is measured with a power meter (PM400K1, Thorlabs Inc., Newton, NJ, USA). Measurements are taken for multiple power values, covering the range of intensities that were registered in previous works from this group [8]. The ability to cover the entire range of powers used for illumination and the deviation from the calibrated values is measured.

#### 2.6.2. Sensitivity to Low Concentrations of ICG

To test the sensitivity of the device in detecting low concentrations of ICG, the output produced by the sensors to the fluorescence intensity from multiple concentrations of ICG was measured. Measurements are taken for the camera and photodetectors in the NIR I and II windows. The minimum concentration that emits a detectable fluorescence is then determined.

The targets are prepared with a mix of homogenized porcine liver and a fresh ICG at a known concentration. Measurements are carried out by placing the tip of the endoscope into the mix.

#### 2.6.3. Sensitivity to Distant Lesions

The sensitivity of the scope in detecting fluorescence from distant lesions in the liver is evaluated by measuring the intensity registered by the scope at several distances from an emitter target embedded in homogenized liver.

The phantom for this experiment consists of a cubic 30 mm quartz cuvette filled with homogenized liver that emulates liver parenchyma. A smaller cuvette filled with a mix of ICG and liver was placed at the bottom to simulate the lesion. This secondary cuvette had a rounded shape, a diameter of 1 cm and a height of 5 mm. The top is covered with a coverslip to avoid contamination of the non-fluorescent homogenized liver while maintaining optimal optical transmission.

The signal measured by the endoscope is recorded while translating it towards the target for a range of 20 mm at very low speed (0.2 mm/s).

#### 2.6.4. Sensitivity to the Presence of Background Fluorescence

This experiment evaluates the effect of the presence of background fluorescence from the parenchyma on the sensitivity of the scope in detecting distant lesions. During liver procedures using ICG, the healthy tissue is expected to clear most of the dye, while the concentration in the tumor remains higher. The remaining ICG in the parenchyma emits a background intensity that affects the ability of the endoscope to detect lesions. The amount of remaining ICG in the parenchyma depends on the elapsed time between the injection and the procedure and on the liver function [16] of the patient.

The ability to detect distant lesions in the presence of background fluorescence was evaluated using the same experimental setup as in the previous section. To simulate background fluorescence, known amounts of ICG were added to the liver parenchyma portion of the phantom, which in this case was prepared using a mix of intralipid solution to improve the homogenization of the medium. ICG background concentrations are calculated as target-to-background ratios with respect to a base concentration of 50 μM for the target. The intralipid mix was prepared according to the literature [17] to mimic the optical properties of liver tissue at 830 nm, aiming for a scattering coefficient of 1.2 mm^−1^ [18].

#### 2.6.5. Temporal Response

The temporal response of the photodiode-based detection module is evaluated by measuring the accuracy of the device in detecting fast variations in the incident intensity. Data recorded by the photodetector are compared with the results from the camera. The illumination is carried out by modulating the laser intensity with half sine waveforms of increasing frequencies (0.1, 0.25, 0.5 and 1 Hz). Light is passed through a diffuser for homogenization. An offset was added to the signal to simulate a minimum amount of background fluorescence.

## 3. Results

### 3.1. Detector Linearity

**Figure 2.**
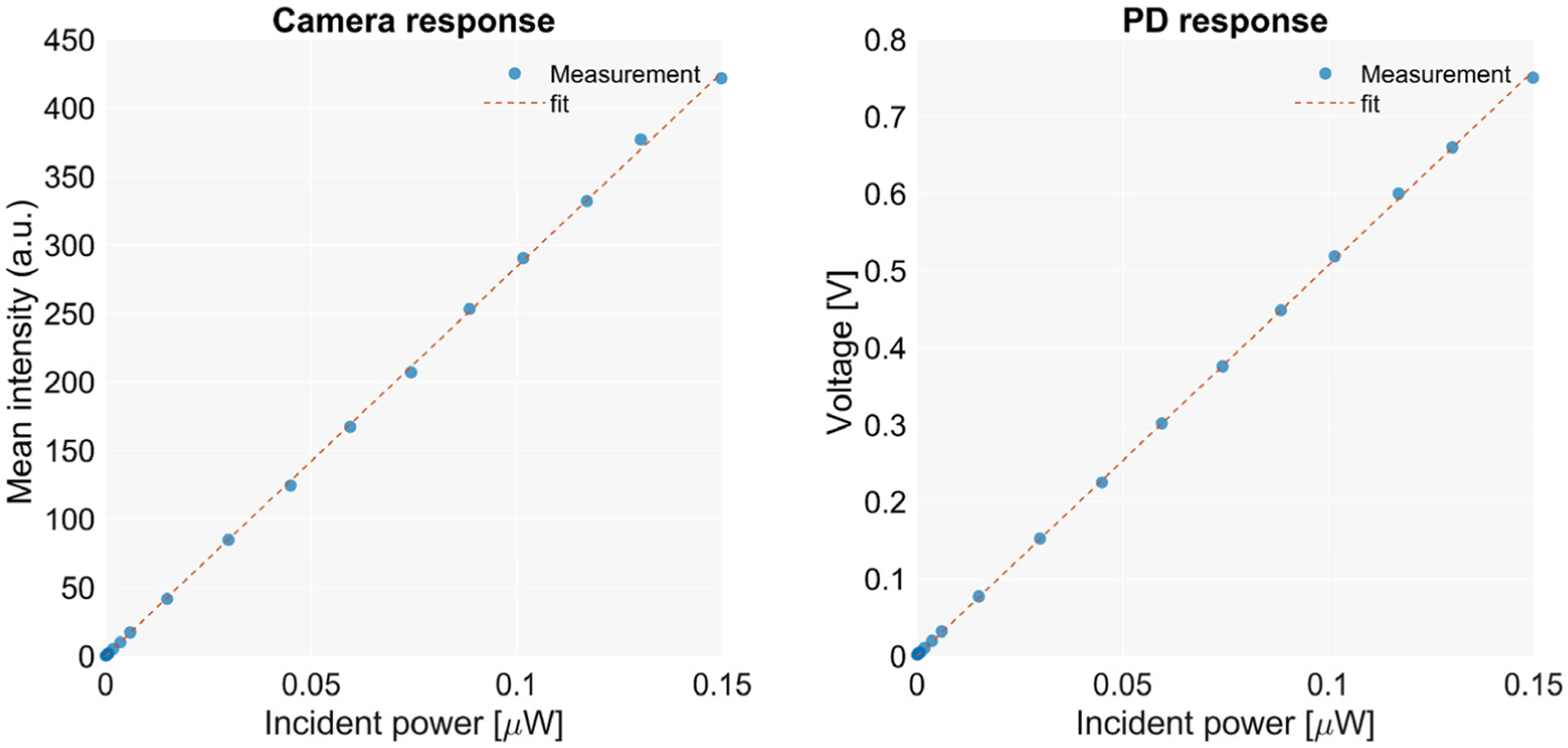
Response of the camera and the NIR I photodetector.

The linear response of the camera and the NIR I photodetector was evaluated by measuring the response of the sensors to incident powers, ranging from 0.15 μW to 0.15 mW, at the back focal plane of the objective and thus on the detector surface. Figure 2 shows the recorded intensities from both devices for each value of the incident power. Either sensor exhibits an adequate linearity, the photodetector being at 32% of its saturation voltage (2.5 V) at the maximum incident power, demonstrating a sufficient dynamic range for this application.

### 3.2. Sensitivity to Low Concentrations of ICG

**Figure 3.**
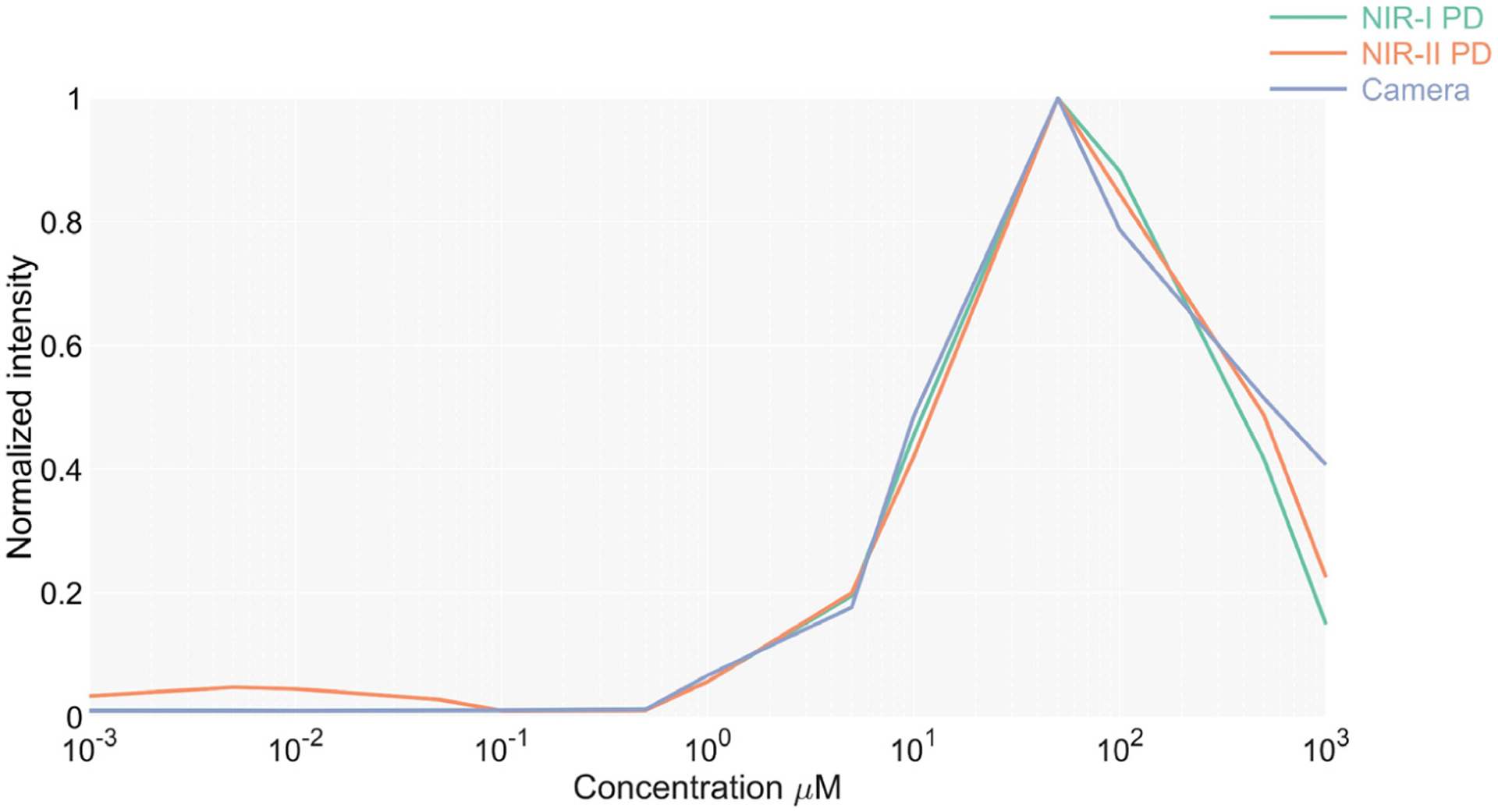
Normalized detected intensities for each sensor at multiple ICG concentrations.

The normalized intensities measured for each concentration of ICG are shown in Figure 3. Both detection technologies registered equivalent normalized intensities and sensitivities, the lowest concentration at which fluorescence was detected being 0.1 μM, which corresponds to 7.74 × 10^−5^ mg/mL. After the intensity peak at 50 μM, the fluorescence intensity starts decaying, matching the data from the literature [19,20]. The ICG fluorescence emission tail in the NIR II can be detected at every concentration, demonstrating the presence of the long NIR emission tail reported by other works [14,15].

### 3.3. Sensitivity to Distant Lesions

**Figure 4.**
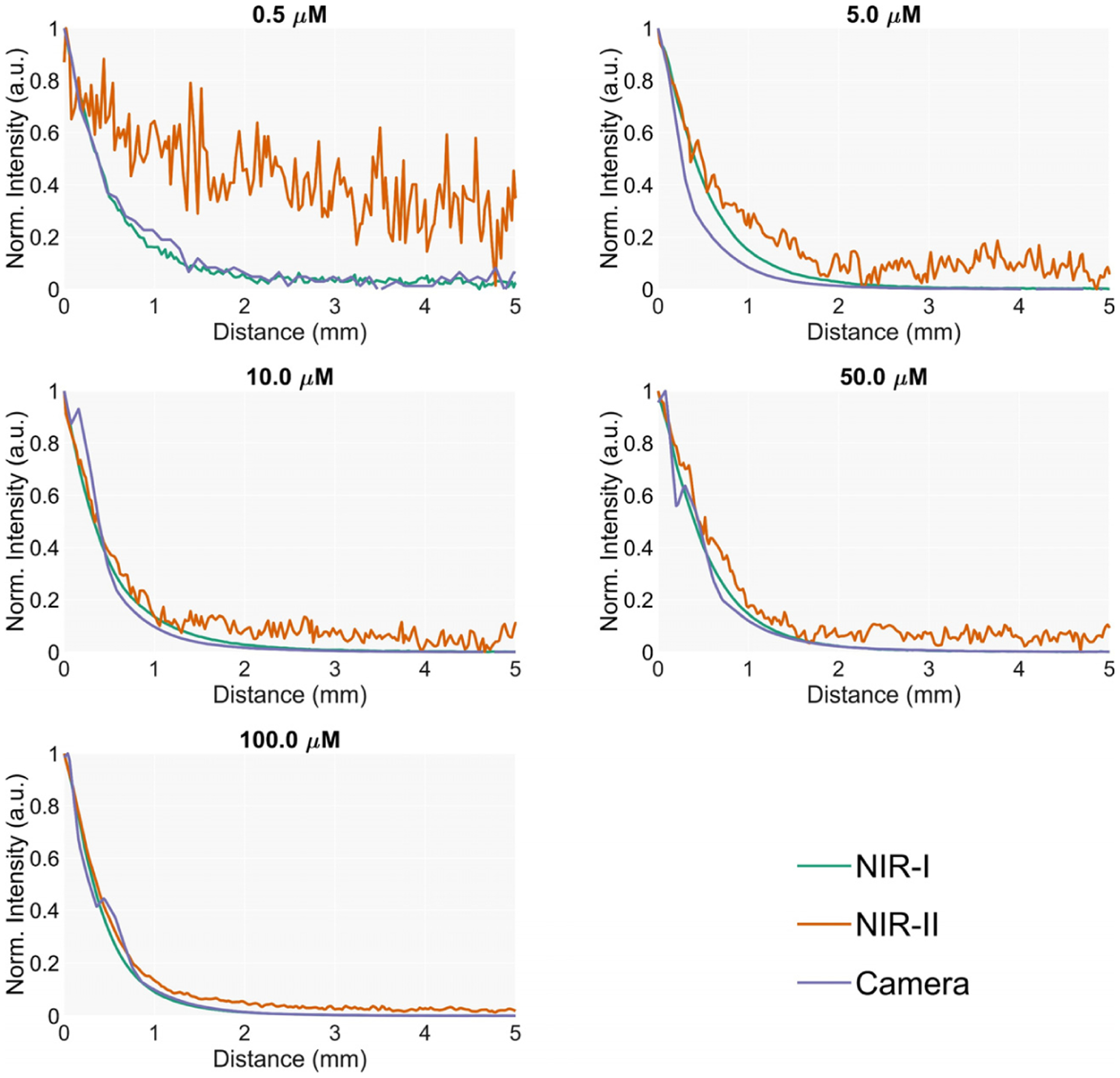
Normalized detected intensity by each sensor versus distance to the lesion for multiple concentrations.

**Figure 5.**
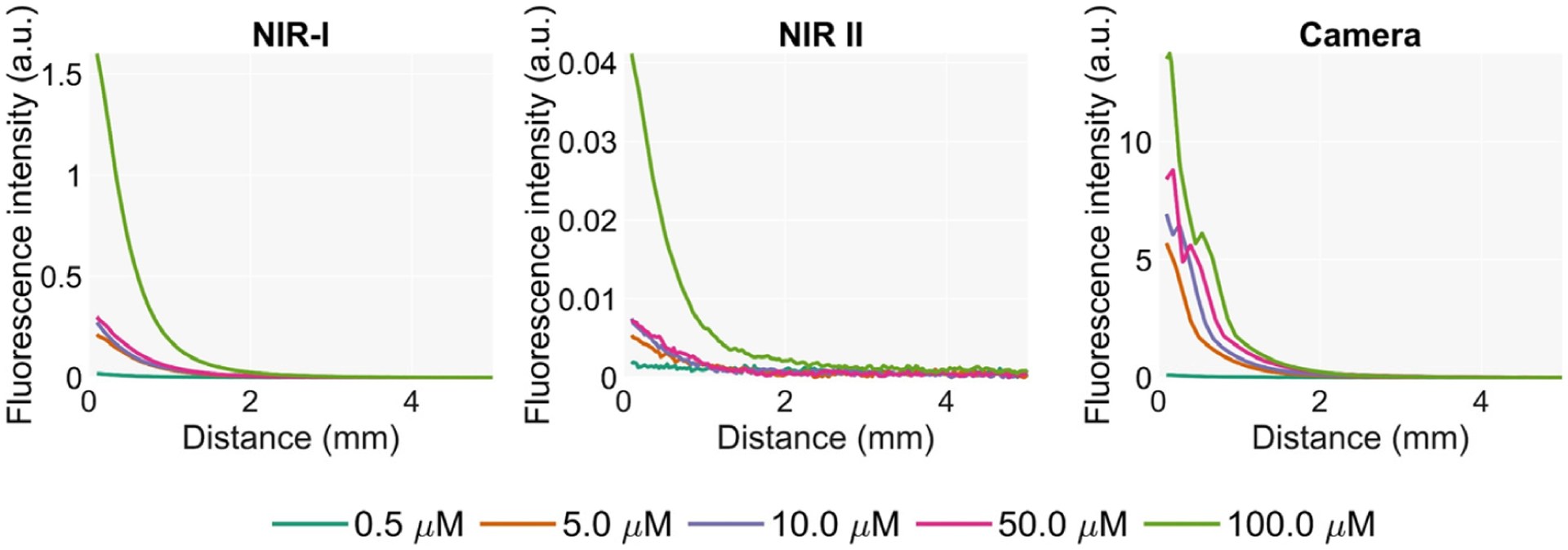
Absolute intensity detected by the sensors versus distance to the lesion for multiple lesion concentrations. The camera device exhibits discontinuities in the slope due to lag of the autoexposure algorithm calculating the integration time while the intensity increases.

The sensitivity of the scope to distant fluorescent lesions was evaluated by translating the endoscope towards the target in the phantom at a very slow speed (0.2 mm/s) in order to minimize trepidations and to allow the camera autoexposure to adjust. The intensity curves showing the change in the detected fluorescence for each position and detector are shown in Figure 4. The NIR I detector and the camera showed similar performances. However, the NIR II detector exhibited a lower signal-to-noise ratio due to the weaker emission of the fluorophore in this region of the spectrum. The distance at which a fluorescence signal increase can be observed, as shown in Figure 5, increases with the concentration of the target being below 2 mm for most of the values, except for 100 μM. The reduced diameter of the endoscope core limits the system’s efficiency in collecting light from a distance, showing significantly smaller detection distances than in works using planar illumination [14].

In Figure 5, the curves for the camera show discontinuities in the slope and inconsistencies in the maximum values. These artefacts are caused by the built-in autoexposure algorithm of the camera. Since the exposure time for every frame is calculated on the intensity values in previous frames, abrupt changes in the intensity produce a biased output that stabilizes when the algorithm accommodates the new intensity. The photodetector-based device showed a much better temporal response, exhibiting a smooth sampling of the increase of the intensity.

### 3.4. NIR I vs. NIR II Signal

In order to evaluate whether the detection of two wavelengths has any potential to retrieve distance information based on the spectral changes of the optical properties of the liver between the NIR I and NIR II windows, we compare the intensity curves measured with the silicon and the InGaAs detectors.

**Figure 6.**
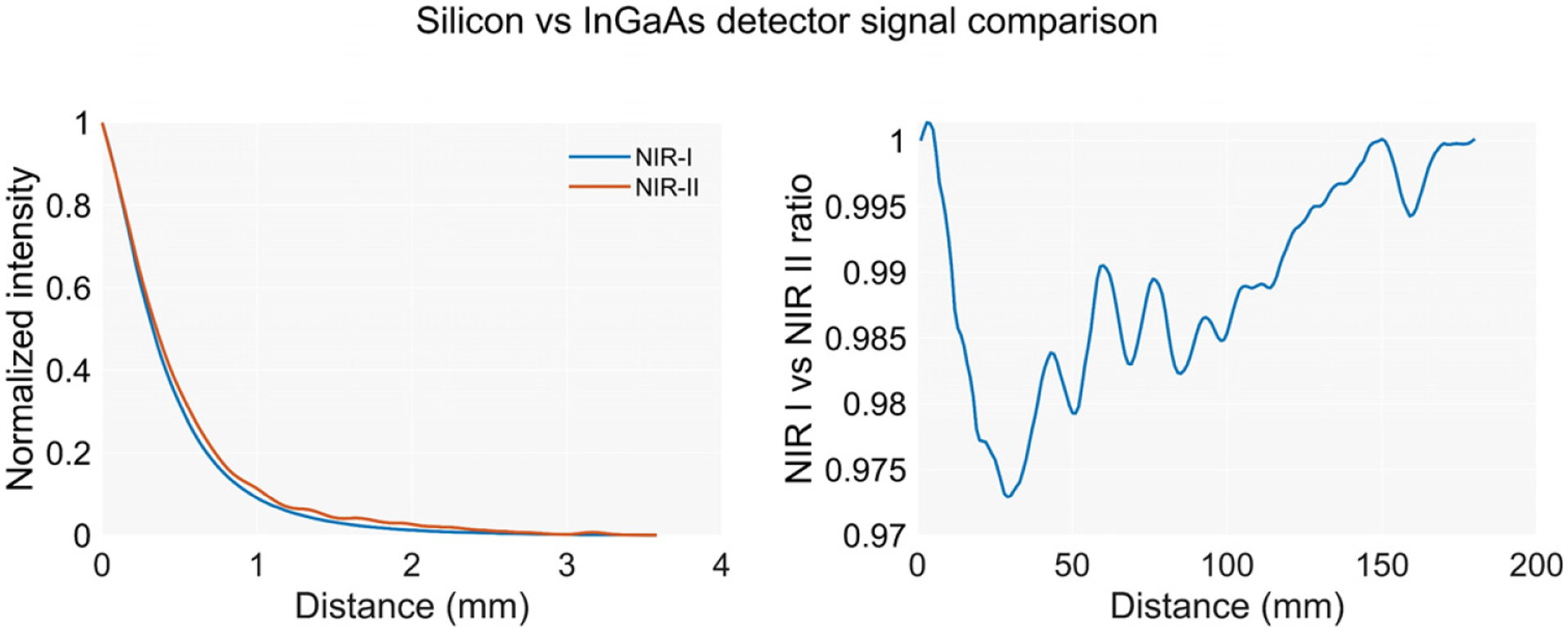
(**Left**) Normalized intensity detected by the NIR I and NIR II detectors while approaching a lesion. (**Right**) NIR I-to-NIR II ratio versus distance to the lesion. The chart shows no significant changes of the ratio other than variations due to noise.

The weaker signal-to-noise ratio of the NIR II signal hinders the comparison for most of the concentrations. Both detectors’ normalized data for the concentration showing the highest emission are plotted in Figure 6. The curves show very similar trends, with ratios very close to 1 for every evaluated distance besides slight arbitrary fluctuations, mostly caused by the noise from the NIR II sensor.

These results suggest that the spectral windows being compared in this experiment, meaning NIR I (830–1000) and NIR-II (1000–1700) nm, may be too broad to register spectral changes in the extinction rate that could potentially provide estimated distance information.

### 3.5. Sensitivity to the Presence of Background Fluorescence

**Figure 7.**
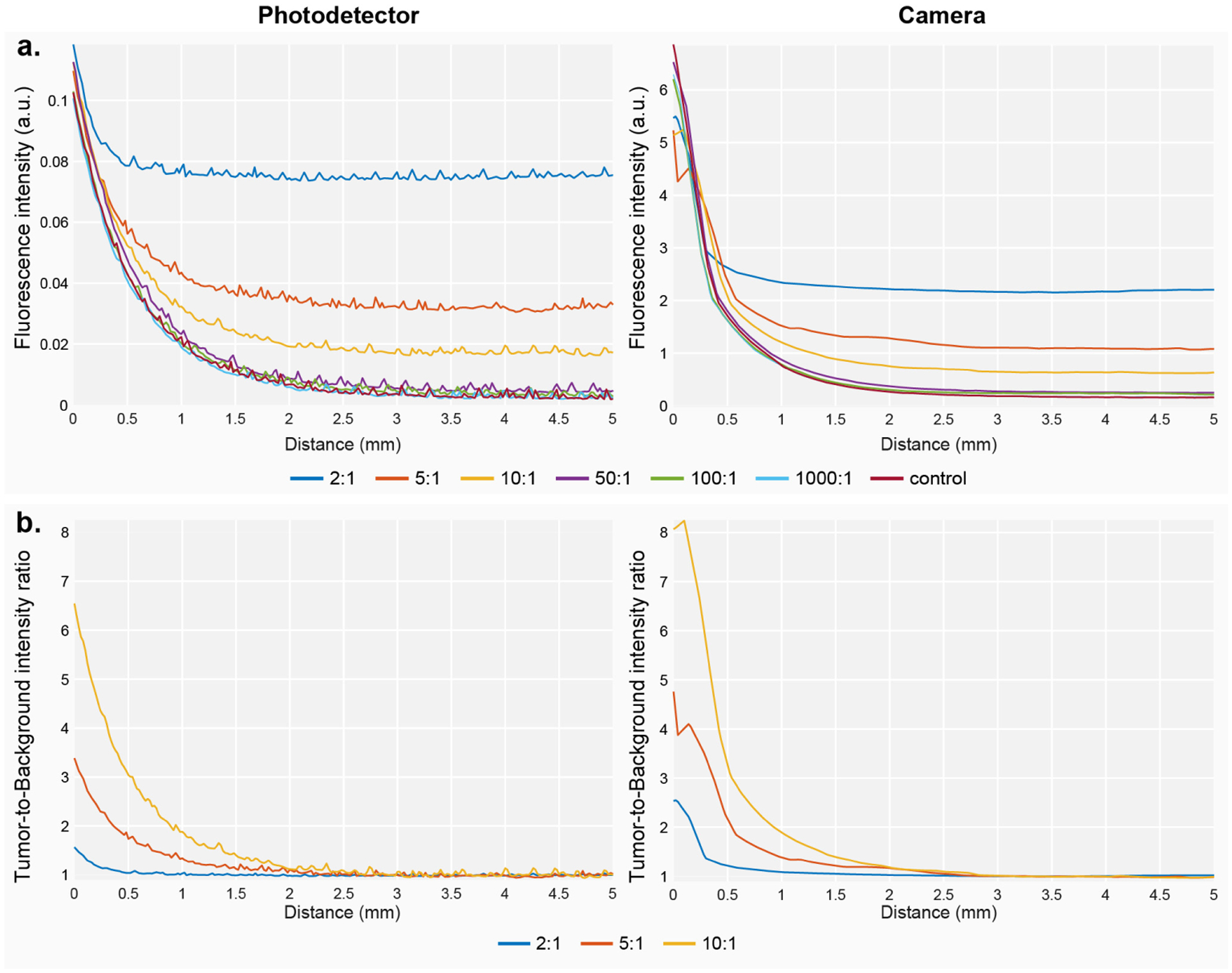
(**a**) Fluorescence intensity at several distances from the lesions for multiple tumor-to-background ratios for both detection modules. (**b**) Fluorescence intensity represented as tumor-to-background ratios detected for multiple background ratios at several distance points from the lesion.

The changes in the sensitivity of the device in the presence of background fluorescence are shown in Figure 7. The data reflect how the system loses detection sensitivity as the concentration of the background increases. For concentration ratios above 50-fold, the effect of the background intensity is negligible. However, below that number, the sensitivity drops dramatically, along with the detectable tumor-to-background ratio.

### 3.6. Temporal Response

**Figure 8.**
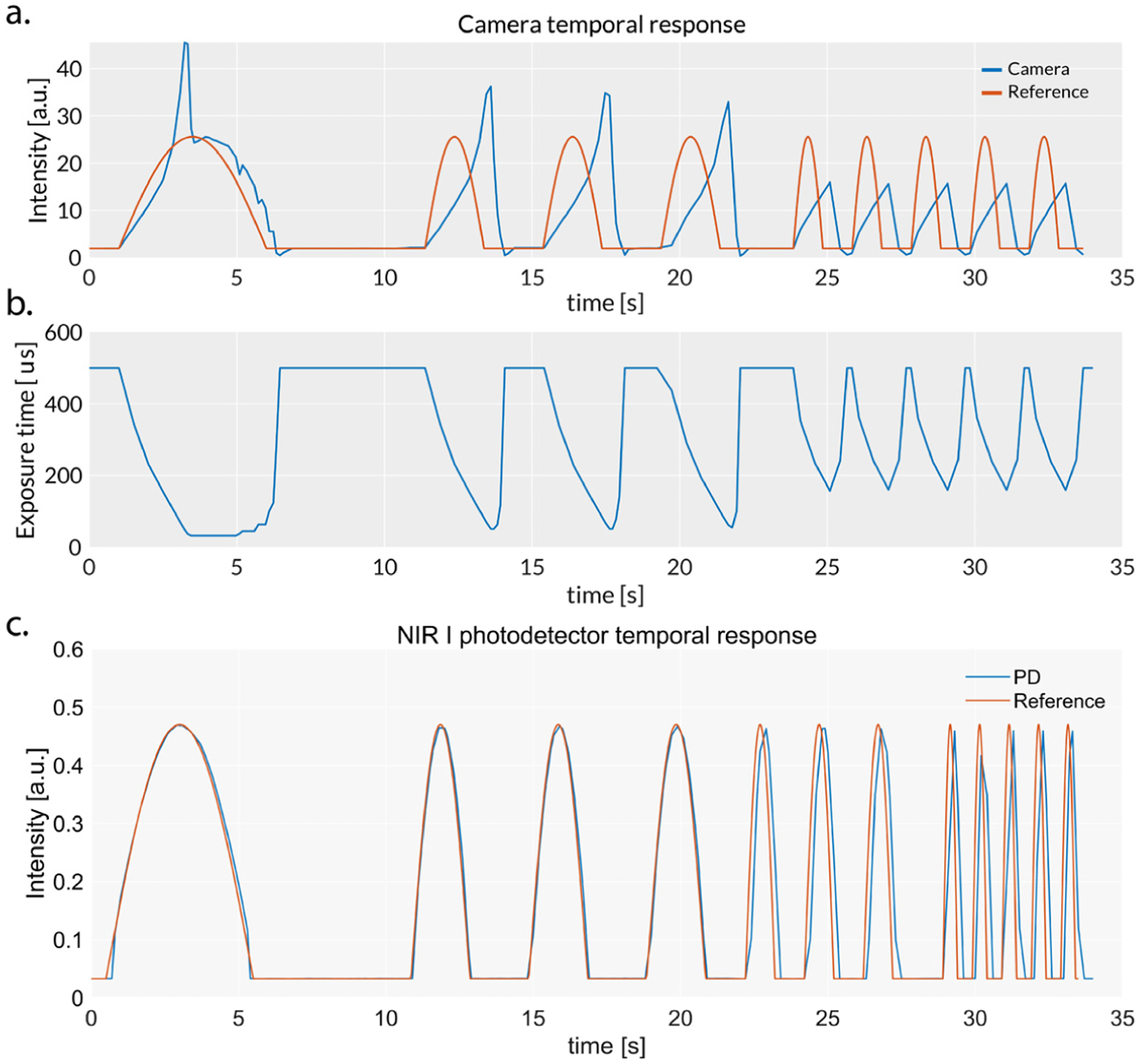
Response of the camera and the photodetector to rapid changes of the incident light intensity. (**a**) Temporal response of the camera. (**b**) Exposure time of the camera determined by the autoexposure algorithm. (**c**) Temporal response of the silicon photodiode.

The photodetector outperformed the camera in terms of registering rapid fluctuations of the intensity (see Figure 8). The accuracy of the camera was very poor, especially when the incident light increased. This is caused by the delay in the response introduced by long exposure times. This can be observed in Figure 8b, where the exposure reaches 500 ms when the incident intensity is minimal. The photodetector showed slight inaccuracies when registering the intensity peaks at 0.5 Hz. This is an under-sampling artifact that could be easily corrected by increasing the sampling rate of the DAQ.

## 4. Discussion

Intraprocedural confirmation of the accurate positioning of a biopsy needle tip at the location of the lesion constitutes critical information during image-guided percutaneous biopsies. Fluorescence detection of ICG that has accumulated in liver tumors is a key milestone in identifying whether the needle is close to a lesion. The fluorescence pattern of lesions cannot be easily predicted by radiological imaging, and thus the detection technology must acquire data with a sufficient temporal resolution and sensitivity to provide reliable information to the radiologist. In this work, we introduce a new version of our endoscope that is capable of detecting light from fluorescent liver tissue with a photodiode through a clinical semirigid endoscope.

The photodetector module showed a comparable sensitivity in detecting both small concentrations of ICG and the fluorescence intensity from deep lesions. In contrast to a camera, this technology had a significantly superior temporal response, demonstrating reliability and accuracy when registering rapid changes in light intensity. Therefore, this device should be considered as a better alternative to recording temporal fluctuations of ICG intensity during biopsy procedures, especially under scenarios where weak fluorescence requires long integration times for the camera.

The results in this manuscript demonstrate that the silicon photodetector outperformed the InGaAs photodiode in terms of detecting ICG fluorescence. Moreover, the combination of measurements in the NIR I and II windows as a ratio did not reveal information about the distance from the tip of the needle to the lesion. The use of more specific spectral windows could potentially enable one to retrieve this information in the future, if the optical properties of the liver are sufficiently different at specific spectral points in such a way that differences are measurable. We believe that this approach might still be complicated to implement, unless the windows are close to the emission peak of ICG or a more sensitive detector technology for detection is used.

Background fluorescence in the liver is shown to be a problematic issue that dramatically reduces the distance threshold for fluorescence detection. Since the endoscope tip is embedded in the medium, background fluorescence prevents the detection of any increase in intensity until the tip is close enough to the lesion to detect an intensity above the background level. For concentration ratios below 50:1, the detected tumor-to-background ratio dropped by up to 3-fold, impeding the detection of fluorescent lesions over up to 1 mm away from the lesion. We believe that the development of spectral methods to distinguish background fluorescence from lesion-emitted fluorescence will be a step towards a more robust detection of tumors during fluorescence-guided biopsies.

## Data Availability

The data presented in this study are available upon request from the corresponding author.
